# Identification of Anoplophora glabripennis (Moschulsky) by its emitted specific volatile organic compounds

**DOI:** 10.1038/s41598-020-61897-0

**Published:** 2020-03-23

**Authors:** Ramona Makarow, Sara Schäfer, Peter Kaul

**Affiliations:** Safety and Security Research Institute, Institute of Detection Technologies, Hochschule Bonn-Rhein-Sieg University of Applied Sciences, von-Liebig-Straße 20, 53359 Rheinbach, Germany

**Keywords:** Chemical biology, Plant sciences

## Abstract

Explorative experiments were done to figure out differences in the emission of volatile organic compounds (VOCs) of not infested trees and trees infested by Anoplophora glabripennis (Asian longhorn beetle, ALB), a quarantine pest. Therefore, VOCs from some native insect species, Anoplophora glabripennis infested Acer, stressed Acer, healthy Acer, Populus and Salix were obtained by enrichment on adsorbents. Qualitative analysis was done by thermal desorption gas chromatography coupled with a mass selective detector (TD-GC/MS). Altogether 169 substances were identified. 11 substances occur from ALB infested or mechanically damaged trees i.e. stressed trees, but not from healthy trees. (+)-Cyclosativene, (+)-α-longipinene, copaene and caryophyllene are detectable only from ALB-infested Acer not from mechanically damaged or healthy Acer. However, these substances are also emitted by healthy Salix. 2,4-Dimethyl-1-heptene is among all tree samples exclusively present in the ambience of ALB-infested trees. It´s rarely detectable from native insect species’ samples.

## Introduction

During the last years the threat through invasive species has increased^1^. The main reasons for a further increase of invasive species in the next years are international trade due to globalisation and climatic changes^1^. Increasing temperatures show an influence on the duration of lifecycle and development of insects. With regards to infested areas in Italy, Bidinger *et al*. expect a shift of infested areas northwards and therefore an extension of infested areas^2^. Concerning Anoplophora glabripennis (ALB), an increase in damage is expected due to its high ability of adaption^2^. Currently, invasive insects’ damage is estimated at US$70.0 billion per year globally and US$3.6 billion per year in Europe. One of the costliest insects is Anoplophora glabripennis with an estimated damage of US$3.0 billion per year in North America and Europe^3^.

Counter measures are visual monitoring of infested areas, pheromone traps and, to some extent, sniffer dogs^4,5^. Sniffer dogs seem to be a good choice especially for major area infestations or major quarantine zones. Besides the use of sniffer dogs for the detection of explosives and for mantrailing^6^, in the last years sniffer dogs have been used for new purposes. They are used for health reasons like the detection of cancer^7–14^ as well as for the search for corpses (human remains detection)^15^, bacteria in milk^16^ or wildlife detection^17^. DeGreef *et al*. were able to show distinct differences concerning the VOC odour profiles of deceased bodies, living human objects and animal remains^16^. The identification of VOCs play an important role for the identification of cancer markers in patients’ breath or liquids^12,14^. Though the use of sniffer dogs still offers a variety of advantages in the detection of odourants^18^, VOCs have become more relevant as instrumental analytical methods, as well as their detection limits, are constantly improved. Influence parameters on the success of a canine’s search are unclear and not completely investigated, however, there is consensus that a copious knowledge of the target’s scent is essential. Thus, more effort has been put into the investigation of volatile organic compounds (VOCs) of the sniffer dog’s target substance, considering a difference in odour between the different sources and an aging process for chemical and biological targets. The knowledge and use of target specific odorous substances can lead to significant improvement in sniffer dog’s training and, as a result, discriminating capacity. The knowledge of target substances is also required for the development of technical detectors based on sensory systems.

Concerning ALB, Makarow *et al*. were able to identify the VOCs emitted by different types of ALB samples (imagoes, larvae and ovipositions)^19^.

The distinction to native insect species and not infested trees were not yet investigated. The analysis of these samples is necessary to figure out specific substances among the 229 identified VOCs. With this work we fill the gap and focus on the determination of the specific ALB volatiles emitted by the quarantine pest ALB. Part of this scope was the development of an approach to extract the specific VOCs, which enable the identification of an ALB infestation in air.

## Materials and Methods

The sampling procedure on adsorption tube from headspace vials and from tree trunks and the analytical methods were carried out in the way Makarow *et al*.^19^ described them. The same instrument was used. However, the main analysis parameters and procedures are mentioned in this section for the sake of completeness.

### Samples

An overview of analysed samples and the used sampling procedure is offered in Table 1. ALB infested trees were measured in five series of measurements, of which two generations of infested trees were analysed (Acer I/Acer II, see Table 2). Acer I-III were measured in a row with one- to two-month delay between the series. Acer II-I and II-II were also measured in a row with a delay of two weeks. The infested trees were located in the quarantine facilities of the Plant Protection Service of Northrhine-Westfalia (PPS NRW) and cultivated under constant greenhouse environment.

**Table 1:** Overview of the analysed samples, the environment of analysis and the sampling procedure including enrichment parameters.

Sample	Sample type	Environment	Sampling method	Enrichment duration, min	Flowrate, ml/min
ALB infested Acer	tree	Greenhouse	on the trunk	90	30
Stressed Acer	tree	Greenhouse	on the trunk	90	30
Zeuzera pyrina infested Populus	tree	open-land	on the trunk	90	30
Saperda cacharias	pupa	laboratory	Headspace-Vial	90	30
	frass	laboratory	Headspace-Vial	90	30
Cossus cossus	larva	laboratory	Headspace-Vial	90	30
Aromia moschata	imago	laboratory	Headspace-Vial	90	30
	larva	laboratory	Headspace-Vial	90	30
Salix (healthy)	tree	open-land	on the trunk	90	30
Populus (healthy)	tree	open-land	on the trunk	90	30
Acer (healthy)	tree	open-land	on the trunk	90	30

**Table 2:** List of substances determined from a TD-GC/MS analysis from ALB-infested Acer.

No.	Class	CAS	Substance	Acer I-I								Acer I- II							Acer I- III								Acer II-I								Acer II-II												Total No. Of 43	Rel.
1	HC	112-40-3	Dodecane	x		x	x	x	x	x	x	x	x		x		x	x	x	x	x	x	x	x		x																					19	44%
2	HC	629-59-4	Tetradecane	x	x	x	x	x		x	x	x	x	x	x		x	x	x			x	x	x		x																					17	40%
3	HC	66-25-1	Hexanal	x		x		x	x	x	x	x	x	x	x		x	x		x								x		x		x															16	37%
4	HC	19549-87-2	2,4-Dimethyl-1-heptene Heptene, 2,4-dimethyl-1-										x	x		x	x	x	x	x	x	x	x	x	x	x									x	x		x									16	37%
5	HC	111-65-9	Octane					x	x	x	x	x	x	x	x		x	x						x		x	x						x										x				15	35%
6	HC	67-64-1	Acetone										x	x					x	x	x	x						x			x		x	x			x						x	x	x		14	33%
7	HC	142-82-5	Heptane							x	x		x		x		x	x		x			x	x		x	x	x		x	x																14	33%
8	HC	589-53-7	Heptane, 4-methyl-										x	x		x	x	x	x		x	x	x	x	x	x									x			x									14	33%
9	HC	124-19-6	Nonanal	x			x	x	x	x	x	x	x	x	x		x	x														x															13	30%
10	HC	629-62-9	Pentadecane	x				x	x	x	x	x	x				x	x	x			x		x		x																					13	30%
11	HC	544-76-3	Hexadecane							x		x		x	x		x	x	x	x		x	x	x	x	x																					13	30%
40	MT	7785-70-8	1R-α-Pinene	x		x	x	x	x	x	x	x	x	x	x		x	x	x	x	x	x	x	x	x	x																					21	49%
41	MT	127-91-3	β-Pinene	x		x	x	x	x	x	x			x	x		x	x					x											x													13	30%
42	MT	13466-78-9	3-Carene									x	x	x	x		x		x	x	x	x	x	x	x	x																					13	30%
43	MT	79-92-5	Camphene	x		x	x	x	x	x	x							x					x						x				x	x													12	28%
44	MT	555-10-2	β-Phellandrene			x	x					x	x	x					x	x	x	x																					x				10	23%
45	MT	5989-27-5	D-Limonene									x	x	x			x	x	x	x		x												x													9	21%
55	ST	22469-52-9	(+)-Cyclosativene	x		x	x	x	x	x	x	x	x	x	x		x	x	x	x			x	x	x	x			x				x	x									x	x	x	x	26	60%
56	ST	3856-25-5/17699-14-8	Copaene/α-Cubebene	x		x	x		x	x	x	x	x	x	x		x	x					x										x	x							x		x	x		x	19	44%
57	ST	5989-08-02	(+)-α-Longipinene	x			x				x	x		x	x		x	x					x	x										x													11	26%
63	Benz	71-43-2	Benzene	x			x	x	x	x	x	x	x	x	x		x	x					x			x				x		x															16	37%
64	Benz	108-88-3	Toluene					x	x	x	x		x	x	x		x	x					x		x	x	x		x			x		x													16	37%
65	Benz	644-30-4	Benzene, 1-(1,5-dimethyl-4-hexen-1-yl)4-methyl-	x								x	x	x				x															x										x	x	x	x	10	23%

Only substances that occur in at least 20% of all measurements are shown. The substances are sorted by class and rate. The numbering in the first column is according to the complete substance-list, which can be seen in the supplemental materials. The substances are sorted by class (HC hydrocarbons, MT monoterpens, ST sesquiterpenes, Benz benzoic substances) and rate (‘total no. of 43’). A cross (x) indicates the occurence of a substance.

Trees of the type Acer without ALB infestation but with mechanical damage (cutting of the branches) and stress (insufficient light and water supply, ‘non-ALB stressed trees’) were measured in three series, they were also located in the facilities of the PPS NRW and cultivated under constant greenhouse environment.

ALB infested Acer and non-ALB stressed Acer were cultivated indoors with artificial light supply and constant temperatures between 20 and 25 °C and a photoperiod of 12 h/12 h light/dark.

**Figure 1:**
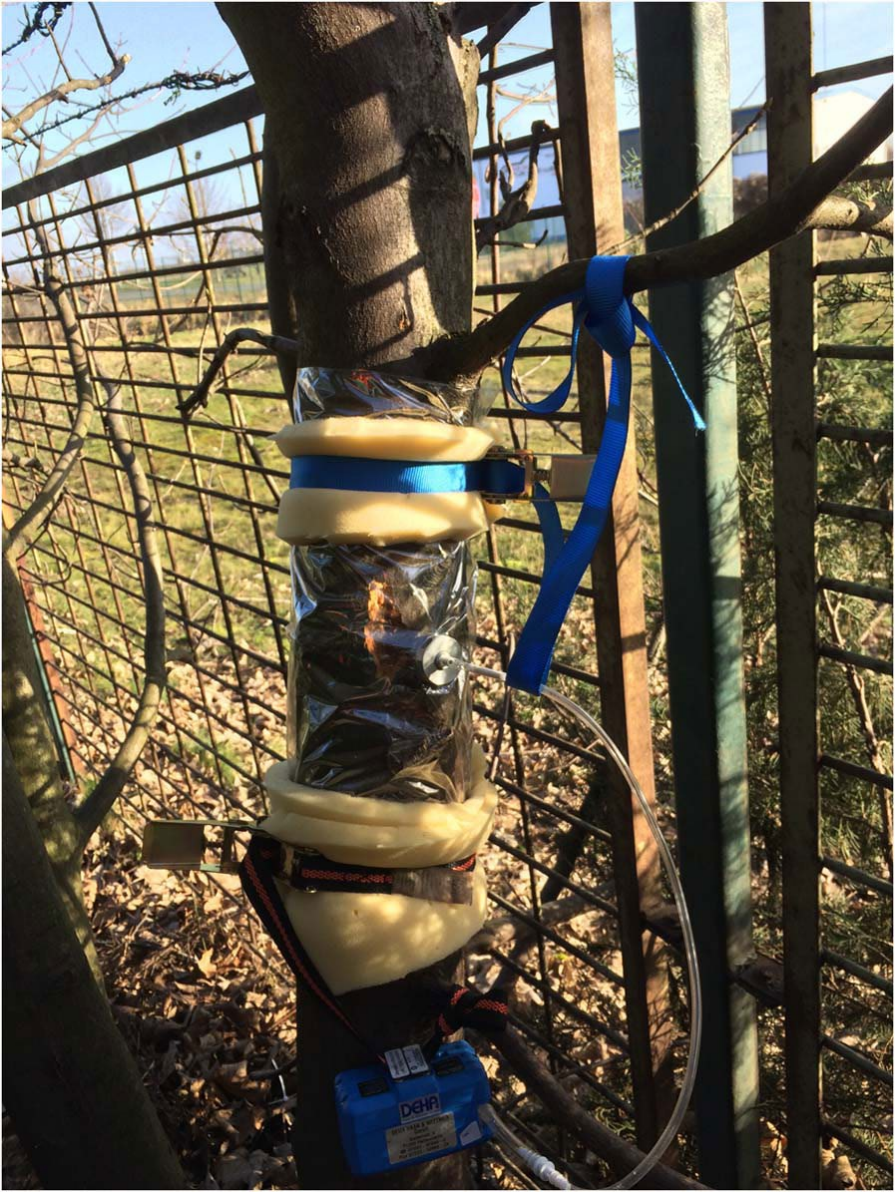
Sampling procedure on a tree trunk. The analysed part is wrapped in Nalophan foil, closed with staples and tension belts.

**Figure 2:**
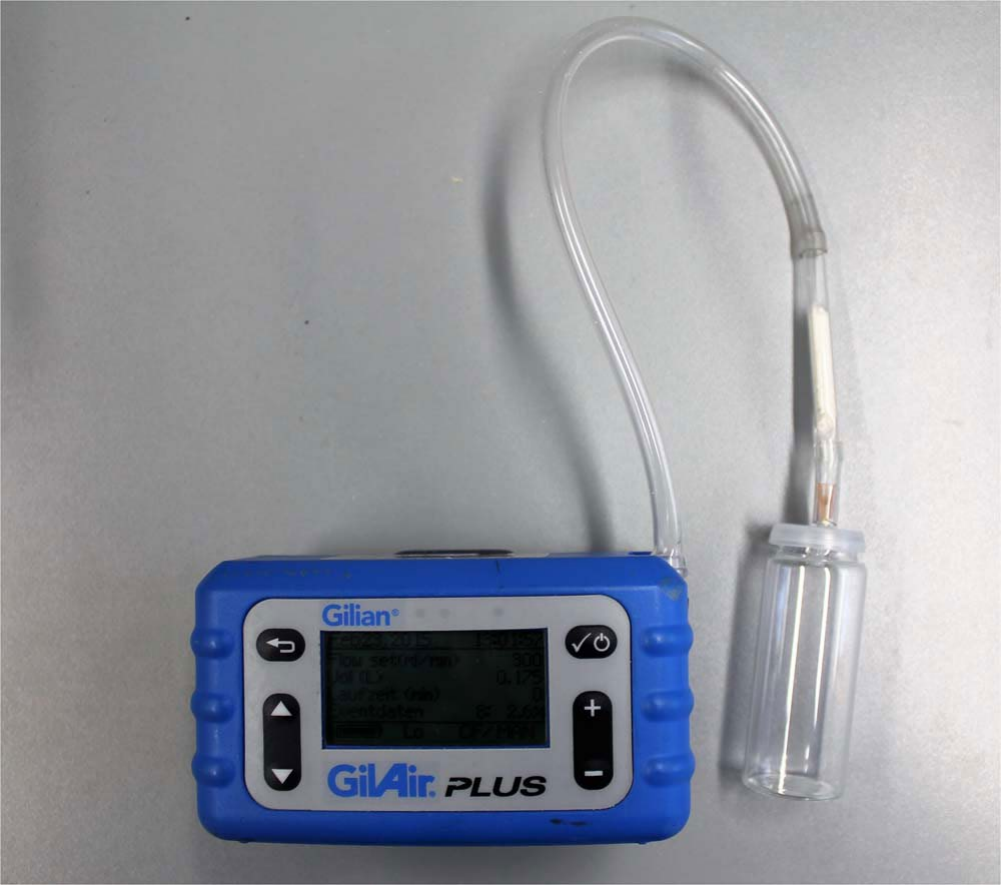
Sampling procedure for beetles, pupae and frass. The samples are stored in the headspace vial while sampling with the pump.

Native insect species samples were *Zeuzera pyrina* infested Populus on open land (sampling method was via the foil wrapped trunk see Fig. 1), *Saperda cacharias* pupa, *Aromia moschata* imago and larva and frass from *Cossus cossus* larva (sampling method see Fig. 2).

As a reference, healthy trees of the types Populus, Acer and Salix were analysed in open field. Open land measurements were carried out as open land offers the best environment for healthy trees. The three types are the most common host trees of ALB in Europe^20^.

### Sampling procedures

Alive beetles and larvae and frass were put into a 20 mL headspace glass vial for the duration of the enrichment of the VOCs on adsorbents. Two small cuts in the septum were used for the enrichment, the pump was adjusted at one hole so that the ambient air was carried over the sample onto the adsorbents tube (see Fig. 2).

The healthy, the ALB infested trees and the non-ALB stressed trees were analysed directly on the trunk (see Fig. 1). Therefore, the sampling method described by Makarow *et al*. was used as the same sampling method was applied^19^. Makarow *et al*. use Nalophan foil for wrapping and staples and tension belts for closing the foil. A self-built adapter is used to adapt the adsorbents tube on the foil. A pump is used to lead the VOCs from the trunk to the adsorbents tube.

For all samples the enrichment time was 90 minutes; flow rate was 30 mL/min. Pumps of two different types were used, namely Gilian GilAir Plus and Gilian LFP-113DC Low Flow Sampler. The flow for the Gilian LFP was adjusted by the Analyt-MTC mass-flow-meter (Analyt-MTC Messtechnik GmbH, Germany). After the enrichment, the adsorbent tubes were immediately stored at −18 °C in a mobile freezer for a maximum of 24 h. Afterwards, the tubes were analysed by TD-GC/MS^19^.

### Conditioning of adsorption tubes

120 mg of adsorbents Tenax TA or Tenax GR were filled into glass tubes (6.0 cm ×0.5 cm) and fixed in place by glass wool. Freshly-filled adsorbent tubes were conditioned once by five-times elution with an acetone/water (90/10) solution, drying at 50 °C for 24 h and placing them three times into an oven at 280 °C, each 1 h, while carbon filtered N_2_ (5.0) with a constant flow was transferred through the tubes. For reconditioning, tubes were placed into an oven at 280 °C for 1 h while carbon filtered N_2_ of constant flow was carried through the tubes. The efficiency of the procedure was controlled by blank analysis. The method was first described by Makarow *et al*.^19^.

Enrichment duration of the volatiles was 90 minutes and enrichment flow was 30 ml/min. Tenax TA and Tenax GR were used for the enrichment of volatiles for the measurements in this paper.

### Instruments

The chromatographic analysis was performed using a 7890A/5975C inert XN MSD GC/MS device (Agilent Technologies) coupled to a thermal desorption unit from Gerstel. The GC was equipped with a DB5-MS capillary column from J&W (30 m × 0.250 mm; 0.25 µm). Helium 5.0 was used as carrier gas and the inlet pressure was 9.1473 psi, which corresponds to a flow of 1.2 mL/min.

### Chromatographic analysis

According to Makarow *et al*. the Cooled Injection System (CIS) was cooled to −120 °C then heated to 250 °C at 12 °C/min and held for 3 minutes. The thermodesorption unit had an initial temperature of 30 °C and was heated to 230 °C at 40 °C/min and held for 1 minute. The transfer temperature was 240 °C. The desorption mode was splitless^19^.

The GC temperature program was held for 2 minutes at 35 °C, then increased to 170 °C at 8 °C/min, then to 240 °C at 60 °C/min and finally held for 2 minutes. Altogether the analysis was carried out with a 1:15 split. Liner temperature was kept to 250 °C^19^.

The complete desorption of VOCs from the adsorbents by the described method was tested by the double analysis of some samples. The double analysis was performed in five time repetitions as part of the development of the analytical method and randomly during the sample analysis stage. The second analysis led to the same results as blanks. Thus it can be expected that the method leads to a total desorption of the analytes from the adsorbents.

The mass spectra were recorded in the electron-impact mode (70 eV) from 30 to 400 DA. Each peak in the chromatogram was identified by comparing the fragmentation pattern typical of each compound to the National Institute of Standards and Technology (NIST) 5.0 database. Only substances with a minimum of 80% match and reproducible retention times in at least three measurements were considered as unambiguous substance identification^19^. The substances 2,4-dmethyl-1-heptene, (+)-α-longipinene, (+)-cyclosativene and (−)-trans-caryophyllene were qualified by standards.

### Chemicals and adsorbent material

For the conditioning of the adsorbents tubes, acetone of HPLC grade (Rotisolve, Carl Roth GmbH + Co KG, (Germany)) was used. The water was self-purified by a MilliQ System. For the enrichment Tenax TA (mesh 60/80) and Tenax GR (mesh 20/35) (Alltech Associates Inc.; Buchem BV (Netherlands)) and silanized glass wool (Sigma Aldrich (Germany)) were used. For tubing Tygon tubes (Carl Roth GmbH + Co KG (Germany)) were used. The Nalophan foil from Kalle GmbH (Germany) was used for wrapping.

As standards 2,4-dimethyl-1-heptene (CAS 19549-87-2, 95%) from Combi-Blocks, (+)-α-longipinene (CAS 5989-08-2, ≥99% sum of enantiomers) from Aldrich, (+)-cyclosativene (CAS 22469-52-9, 99%) from Sigma-Aldrich and (−)-trans-caryophyllene from Sigma-Aldrich (all purchased from Merck (Germany)) were used. The standards were dissolved in methanol (Rotisolve ≥ 99.98%; Carl Roth GmbH + Co KG (Germany)). A volume of 50 µl, which corresponds a mass of 100 ng of each standard, were injected on the adsorbents with a microliter syringe (Hamilton). The solvent was dried by pumping ambient air with a flow of 400 ml/min for 4 minutes over the adsorbents tube. In order to exclude contaminations, the drying procedure was carried out with blank adsorbents.

## Results

**Figure 3:**
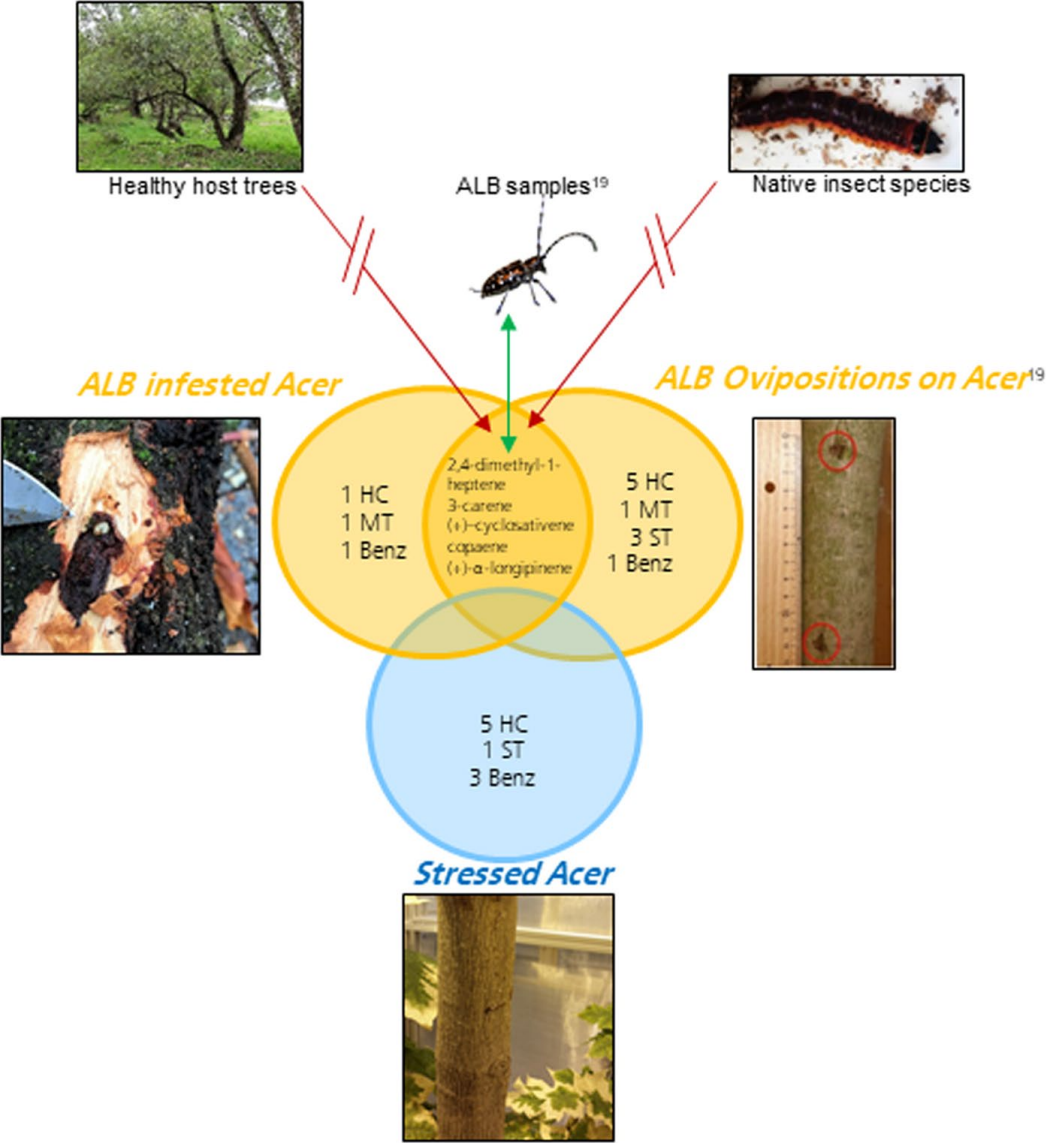
Scheme of extracting the specific volatile organic compounds emitted from Anoplophora glabripennis including the analysis carried out by Makarow *et al*.^19^. The green arrow indicates the overlap of substances to the substances detected from standalone ALB samples. The red arrows indicate that no overlap of substances of healthy trees as well as of native insect species is requested.

With the scope of determining the specific volatiles of Anoplophora glabripennis the following analysis were carried out: when considering the stress induced change of VOC pattern of a plant, the first step was the analysis of ALB infested Acer (with larvae inside and with fresh ovipositions) and non-ALB stressed Acer. The VOCs deriving from the two types of ALB infested Acer, but not detectable from non-ALB stressed Acer were considered ALB induced. The comparison of these VOCs with VOCs from single ALB samples (beetles and larvae) published by Makarow *et al*.^19^ underpinned an ALB-VOC pattern. Finally the distinction between VOCs emitted by native insect species as well as healthy trees leads to some ALB-specific volatiles (see Fig. 3).

The detailed results of the native insect species, the healthy trees, the trees with mechanical damage and the trees infested by ALB are given in Table 2 and in the supplementary material. The tables show a list of measured substances (name and CAS number) sorted by class (hydrocarbons (HC), benzoic substances (Benz) and mono- (MT) and sesquiterpenes (ST)). The cross indicates the occurrence of the substance. The tables also show the absolute and relative frequency.

The substances copaene and α-cubebene are listed as one substance, due to the very high similarity especially in the mass spectrum. Neither distinction via MS nor by use of standards was possible, because standards for these sesquiterpenes were not available at all. The occurrence and overlay of both substances is also possible.

### Comparison of ALB-infested Acer (by larvae and by ovipositions) and non-ALB stressed Acer in greenhouse

In Table 2 the substances from ALB-infested Acer, ALB ovipositions on Acer (Makarow *et al*.) and stressed Acer are shown. The analysis of ALB-infested Acer (by larvae) showed 73 substances and with (+)-cyclosativene, one substance that occurs in over 50% of all measurements. Analysis of non-ALB stressed Acer show 27 substances, none of which occur in at least 50% of all measurements. 15 substances were emitted by both types of stressed Acer: 9 hydrocarbons (dodecane, hexanal, octane, pentadecane, acetone, heptane, nonanal, hexadecane and tetradecane), 4 monoterpens (1R-α-pinene, 3-carene, limonene and β-pinene), 2 benzoic substances (toluene and benzene). Compared to the results from Makarow *et al*. from the analysis of ALB ovipositions on Acer under the same greenhouse parameters there are 5 substances, that derive from both, ALB-infested Acer and ALB ovipositions, on Acer but do not derive from non-ALB stressed Acer: 2,4-dimethyl-1-heptene, camphene, (+)-cyclosativene, copaene, (+)-α-longipinene (see Table 3). The analysis of these samples were carried out under comparable environment. The results from Makarow *et al*. listed in the Table were reduced to the substances, that were detectable in both generations of ALB ovipositions.

**Table 3:** Results from the analysis carried out under greenhouse environment with “ALB infested Acer”: substances detected from ALB infested Acer (43 measurements); “ALB ovipositions on Acer”: results from Makarow *et al.* and “stressed Acer”: substances detected from Acer stressed by insufficient water and light supply and cut off branches. Substances are sorted by class with HC hydrocarbons, MT monoterpenes, ST sesquiterpenes and Benz benzoic substances.

Class	CAS	Substance	ALB-infested Acer		ALB Oviposition on Acer*				stressed Acer	
			Total No. Of 43	Rel.	no. total of 86	Rel.	no. of P1	no. of P2	Total No. of 22	Rel.
HC	112-40-3	Dodecane	19	44%	32	37%	16	16	5	23%
HC	66-25-1	Hexanal	16	37%	16	19%	3	13	5	23%
HC	19549-87-2	Heptene, 2,4-Diemethyl-1-	16	37%	12	14%	6	6		
HC	111-65-9	Octane	15	35%	47	55%	25	22	5	23%
HC	629-62-9	Pentadecane	13	30%	24	28%	21	3	5	23%
HC	67-64-1	Acetone	14	33%					3	14%
HC	142-82-5	Heptane	14	33%					3	14%
HC	589-53-7	Heptane, 4-methyl-	14	33%						
HC	124-19-6	Nonanal	13	30%					5	23%
HC	544-76-3	Hexadecane	13	30%					5	23%
HC	629-59-4	Tetradecane	17	40%					5	23%
HC	2213-23-2	Heptane, 2,4-diemethyl-			11	13%	6	5	4	18%
HC	2847-72-5	Decane, 4-methyl-			12	14%	5	7		
HC	565-75-3	Pentane, 2,3,4-trimethyl-			11	13%	8	3		
HC	2216-34-4	Octane, 4-methyl-			9	10%	5	4		
HC	17302-27-1	Nonane, 2,5- dimethyl-			7	8%	4	3		
HC	7045-71-8	Undecane, 2-methyl-			6	7%	3	3		
HC	67-63-0	Isopropyl Alcohol							6	27%
HC	124-18-5	Decane							3	14%
HC	17302-28-2	Nonane, 2,6-dimethyl-							3	14%
HC	1120-21-4	Undecane							3	14%
HC	629-78-7	Heptadecane							3	14%
MT	7785-70-8	1R-α-Pinene	21	49%	32	37%	15	17	5	23%
MT	13466-78-9	3-Carene	13	30%	36	42%	14	22	5	23%
MT	79-92-5	Camphene	12	28%	83	97%	24	59		
MT	5989-27-5	D-Limonene	9	21%					5	23%
MT	127-91-3	β-Pinene	13	30%					4	18%
MT	555-10-2	β-Phellandrene	10	23%						
MT	138-86-3	Limonene			40	47%	8	32		
ST	22469-52-9	(+)-Cyclosativene	26	60%	85	99%	26	59		
ST	3856-25-5/17699-14-8	Copaene/α-Cubebene	19	44%	85	99%	26	59		
ST	5989-08-02	(+)-α-Longipinene	11	26%	86	100%	27	59		
ST	87-44-5	Caryophyllene			71	83%	23	48		
ST	495-60-3	Zingiberene			40	47%	5	35		
ST	489-40-7	a-Gurjunene			20	23%	10	10		
ST	475-20-7	(+)-Longifolene							3	14%
Benz	108-88-3	Toluene	16	37%	26	30%	20	6	5	23%
Benz	644-30-4	Benzene, 1-(1,5-dimethyl-4-hexen-1-yl)4-methyl-	10	23%						
Benz	71-43-2	Benzene	16	37%					4	18%
Benz	24157-81-1	2.6-Diisopropylnaphthalene			32	37%	24	8	3	14%
Benz	108-67-8	Benzene, 1,3,5-trimethyl-			11	13%	7	4		
Benz	719-22-2	2,5-Cyclohexadiene-1,4-dione, 2,6-di-tert-Butyl-							5	23%
Benz	106-42-3	P-Xylene							4	18%
Benz	128-37-0	Butylated Hydroxytoluene							4	18%

### Overlap of ALB-infestation and ALB-samples

Makarow *et al*. analysed the airborne VOCs from ALB larva, imago and oviposition. They pointed out that 2,5-dimethyl-1-heptene and (+)-cyclosativene were present in all three sample types. This work shows that they were also detectable from ALB-infested Acer in 60% and 14%, respectively, of all measurements. Furthermore, copaene was suggested to be a marker for ALB. As 44% of 43 measurements on infested Acers show copaene, this observation can be endorsed considering a high variation in biological samples.

### Comparison to healthy trees in open-land

With regards to the need of detecting ALB in an open land scenario, the chemical background of an open land infestation was analysed. That was realised by the analysis of ALB preferred healthy host trees (Salix, Populus, Acer) in open land in a tree nursery. As a result, 42 substances were detected, 9 (6 hydrocarbons, toluene and 1R-α-pinene and (+)-β-pinene) of them in more than 50% or all measurements.

Compared to the analysis of ALB-infested Acer and non-ALB-stressed Acer 12 substances occur in all three tree-samples: 8 hydrocarbons (heptane, octane, nonanal, dodecane, tetradecane, pentadecane, hexadecane, heptadecane), 2 monoterpenes (1R-α-pinene, β-pinene) and 2 benzoic substances (benzene, toluene). 11 substances (6 hydrocarbons, 3-carene and 4 benzoic substances) were not detectable from healthy trees, but from the group of stressed trees (from ALB-infestation and non-ALB stress).

The three types of healthy trees (Acer, Populus, Salix) show significant differences in their emission of VOCs, especially with regards to sesquiterpenes. With the exception of (−)-alloaromadendrene, which only occurs from Populus and sesquiterpenes, which only derive from Salix. Among them are copaene, (+)-cyclosativene and caryophyllene with an occurrence of 100%, 78% and 67%, respectively, in Salix measurements.

It is also noticeable that healthy trees show a stable emission of octane (100% for Acer, 73% for Populus and 100% for Salix) that reduces in case of stress (35% for ALB-infested Acer, 55% for Acer with ALB oviposition^19^, 23% for non-ALB stressed Acer).

### Distinction ALB and other VOC-Sources

To ensure the detected VOCs originate from the ALB infestation and not from other insect species, the analysis of some native insect species were carried out. The analysis of some native species shown in the supplemental material (species and its sample type) result in altogether 27 substances: 20 hydrocarbons, 6 benzoic substances and, with (+)-longifolene, one sesquiterpene. With regard to the VOC-emission of ALB-infested trees and standalone ALB-material from Makarow *et al*., Table 4 shows that native species, as well as all ALB samples, emit 2,4-dimethyl-1-heptene and dodecane.

**Table 4:** List of substances that occur in at least 50% of one of the ALB-batches in comparison to other possible VOC sources. Starred data originates from Makarow *et al*.^19^.

Class	CAS	Substance	VOCs from ALB-Sources				Other Sources				
			Infested tree	Oviposition*	Larva*	Beetle*	Healthy tree, open land			Mechanically damaged tree	Native
							Acer	Populus	Salix		
ST	100010-98-1	(+)-Cyclosativene	60	99	91	23	—	—	67	—	—
ST	5989-08-02	(+)-α-Longipinene	26	100	14	—	—	—	56	—	—
ST	3856-25-5/17699-14-8	Copaene/α-Cubebene	44	99	77	31	—	9	89	—	—
ST	87-44-5	Caryophyllene	9	83	—	62	—	—	78	—	—
HC	111-65-9	Octane	35	55	—	—	100	73	100	23	—
HC	19549-87-2	2,4-Dimethyl-1-heptene	37	6	59	69	—	—	—	—	16
HC	112-40-3	Dodecane	44	16	50	62	43	—	33	23	26
MT	138-86-3	Limonene		32	95	—	14	—	22		—
MT	7785-70-8	1R-α-Pinene	49	17	32	100	43	100	100	23	—
MT	13466-78-9	3-Carene	16	22	—	54	—	—	—	23	—
MT	79-92-5	Camphene	28	97	—	—	14	36	89	—	—

An overview of the most relevant substances with a reliable occurrence over all sample types is given in Table 4. That table shows the comparison between ALB-samples and non-ALB-samples.

## Discussion

This work showed that the identification of *Anoplophora glabripennis* (Moschulsky) by its emitted specific volatile organic compounds is possible. The approach of extraction volatiles by comparison of infested trees, non-ALB stressed trees, ALB samples in different development stages, native species and healthy trees enabled the successful identification of the specific ALB VOCs.

Based on the results of Makarow *et al*. that copaene in combination with (+)-cyclosativene and 2,4-dimethyl-1-heptene hint at ALB, this work showed that these ALB originated VOCs are also present and detectable in the ambient air of a living tree that is infested with ALB.

Concerning samples that were taken right on a trunk, we were able to show that the named VOCs do not origin from the stress of a tree in general, as cut and undersupplied Acers do not show these VOCs. But copaene and (+)-cyclosativene are emitted by healthy Salix as well as (+)-α-longipinene and caryophyllene. Healthy Acer and Populus did not or rarely show these substances. Qualitatively an interference between ALB-infested trees and Salix is possible.

Makarow *et al*. showed with a literature research that the named substances were not yet detected from insects. For a more reliable exclusion of cross sensitivities we were able to show that there was no detectable emission of copaene and (+)-cyclosativene among some native species. Whereas 2,4-dimethyl-1-heptene was detectable from *Saperda cacharias* pupa and *Cossus cossus* larva.

Although, 44% occurrence of copaene in infested trees seems to be of moderate statistical proof, the biological variance needs to be taken into account. Among all 23 measurements of Acer-I the occurrence of copaene is with 57% almost double compared to all Acer-II measurements with 30% occurrence. As this occurrence gap can be observed over all emitted VOCs (286 in all 23 Acer-I analysis; 55 in all 20 Acer-II analysis) it´s most likely that Acer-II was analysed under different biological environment than Acer-I, although the parameters of light, temperature and humidity were kept constant. As the influences on trees’ VOC emission is not yet known it is not possible to control or predict the biological sample and its environmental impacts. By contrast, there is no occurrence of copaene at all for non-ALB stressed Acer or native insect species.

To sum it up, the presence of copaene, (+)-cyclosativene and (+)-α-longipinene gives a very strong hint to an ALB-infestation especially if the suspicious tree is not of the type Salix. If the host tree is of the type Salix, then the addition of 2,4-dimethyl-1-heptene and 3-carene could specify the VOC pattern toward ALB. Considering the high biological variance of VOC emission multiple measurements of a suspicious tree must be acquired to clarify an ALB-infestation without cutting off the tree.

These findings offer the basis for the development of early stage detection technologies of quarantine pests. The clarification of airborne substances offers the opportunity of an improved detection due to better availability and accessibility in air than for physical investigation methods e.g. DNS-sequencing. This is particularly relevant as the threat of invasive alien species is predicted to aggravate in the future^3^.

## Supplementary information

Supplementary information.

## Data Availability

The data generated and analysed during the current study are included in this published article and its Supplementary Information Files.

## References

[1] Acosta AL, Giannini TC, Imperatriz-Fonseca VL, Saraiva AM (2016). Worldwide Alien Invasion: A Methodological Approach to Forecast the Potential Spread of a Highly Invasive Pollinator. PLoS one.

[2] Kerstin, B. *Schadpotenzial gebietsfremder, invasiver Käferarten unter Berücksichtigung des globalen Klimawandels und rechtlicher Aspekte* (2012).

[3] Bradshaw CJA (2016). Massive yet grossly underestimated global costs of invasive insects. Nat. Commun..

[4] Trotter RT, Keena MA (2016). A Variable-Instar Climate-Driven Individual Beetle-Based Phenology Model for the Invasive Asian Longhorned Beetle (Coleoptera: Cerambycidae). Environ. entomology.

[5] Xu T (2017). Identification of a male-produced sex-aggregation pheromone for a highly invasive cerambycid beetle, Aromia bungii. Sci. Rep..

[6] Marchal, S., Bregeras, O., Puaux, D., Gervais, R. & Ferry, B. Rigorous Training of Dogs Leads to High Accuracy in Human Scent Matching-To-Sample Performance. *Plos one* (2016). 10.1371/journal.pone.0146963 PMC4749222 26863620

[7] Boedeker, E., Friedel, G. & Walles, T. Sniffer dogs as part of a bimodal bionic research approach to develop a lung cancer screening. *Interactive CardioVascular and Thoracic Surgery*, 511–514 (2012). 10.1093/icvts/ivr070 PMC3329290 22345057

[8] Elliker, K. R. *et al*. Key considerations for the experimental training and evaluation of cancer odour detection dogs: lessons learnet from a double-blind, controlled trial of prostate cancer detection. *BMC Urology* (2014). 10.1186/1471-2490-14-22 PMC3945616 24575737

[9] Guerrero-Flores H (2017). A non-invasive tool for detecting cervical cancer odor by trained scent dogs. BMC cancer.

[10] Hackner K (2016). Canine scent detection for the diagnosis of lung cancer in a screening-like situation. J. Breath. Res..

[11] Concetta, P. *et al*. Cancer sniffer dogs: how can we translate this peculiarity in laboratory medicine? Results of a pilot study on gastrointestinal cancers. *Clinical Chemistry and Laboratory Medicine*, 56, https://www.degruyter.com/view/j/cclm.2018.56.issue-1/cclm-2016-1158/cclm-2016-1158.xml. 10.1515/cclm-2016-1158 28590915

[12] Schallschmidt K (2016). Comparison of volatile organic compounds from lung cancer patients and healthy controls—challenges and limitations of an observational study. J. Breath. Res..

[13] Seo I-S (2018). Cross detection for odor of metabolic waste between breast and colorectal cancer using canine olfaction. PLoS one.

[14] Willis CM, Britton LE, Harris R, Wallace J, Guest CM (2010). Volatile organic compounds as biomarkers of bladder cancer: Sensitivity and specificity using trained sniffer dogs. Cancer biomarkers: Sect. A Dis. markers.

[15] DeGreeff, L. E. & Furton, K. G. Collection and identification of human remains volatiles by non-contact, dynamic airflow sampling and SPME-GC/MS using various sorbent materials. *Analytical and Bioanalytical Chemistry*, 1295–1307 (2011). 10.1007/s00216-011-5167-0 21695377

[16] Fischer-Tenhagen C, Theby V, Krömker V, Heuwieser W (2018). Detecting Staphylococcus aureus in milk from dairy cows using sniffer dogs. J. Dairy. Sci..

[17] Mills G (2018). Sniffer dogs to help combat wildlife crime. Veterinary Rec..

[18] Furton KG, Caraballo NI, Cerreta MM, Holness HK (2015). Advances in the use of odour as forensic evidence through optimizing and standardizing instruments and canines. Philos. Transactions: Biol. Sci..

[19] Makarow R (2019). Investigation of volatile organic compounds emitted by Anoplophora glabripennis (Moschulsky) using thermal desorption and gas chromatography-mass spectrometry. Microchemical J..

[20] JKI, Institut für nationale und internationale Angelegenheiten der Pflanzengesundheit. Notfallplan und Leitlinie zur Bekämpfung von Anoplophora glabripennis in Deutschland. *Bundesanzeiger*, 1–82 (2016).

